# A V-Band Wideband Power Amplifier with High Gain in a 130 nm SiGe BiCMOS Process

**DOI:** 10.3390/mi15091077

**Published:** 2024-08-26

**Authors:** Jianing Hu, Jialong Wan, Yi Shen, Wei Zhao, Jiang Luo

**Affiliations:** 1 School of Electronics and Information, Hangzhou Dianzi University, Hangzhou 310018, China; 2 School of Microelectronics, South China University of Technology, Guangzhou 511442, China; 3 State Key Laboratory of Millimeter Waves, Southeast University, Nanjing 210096, China

**Keywords:** millimeter-wave (mm-wave), SiGe BiCMOS, bandwidth extension, power amplifier

## Abstract

This paper introduces a high-gain wideband power amplifier (PA) designed for V-band applications, operating across 52 to 65 GHz. The proposed PA design employs a combination of techniques, including pole-gain distribution, base-capacitive peaking, and the parallel configuration of multiple small-sized transistors. These strategies enable significant bandwidth extension while maintaining high gain, substantial output power, and a compact footprint. A two-stage PA using the combination technique was developed and fabricated in a 130 nm SiGe BiCMOS process. The PA prototype achieved a peak gain of 27.3 dB at 64 GHz, with a 3 dB bandwidth exceeding 13 GHz and a fractional bandwidth greater than 22.2%. It delivered a maximum saturated output power of 19.7 dBm and an output 1 dB compression point of 18 dBm. Moreover, the PA chip occupied a total silicon area of 0.57 mm^2^, including all testing pads with a compact core size of 0.198 mm^2^.

## 1. Introduction

The V-band encompasses a rich spectrum of 40 to 75 GHz and has been widely utilized in high-speed wireless communications, high-resolution radar, and radio telescopes in recent years [1,2,3,4]. These applications have driven an increasing demand for multi-band, wideband millimeter-wave (mm-wave) front ends. Undoubtedly, the power amplifier (PA) plays a crucial role as an indispensable component in a mm-wave transmitter. It is expected to provide wide bandwidth, high gain, high output power (P_out_), and excellent linearity while maintaining a flat frequency response within the target band, ensuring optimal spectrum efficiency. Due to the effective scaling of device sizes, SiGe BiCMOS has proven to be an efficient technology for mm-wave systems [5,6,7]. Moreover, its compatibility with bulk silicon CMOS for biasing circuits and digital logic makes it well suited for cost-sensitive mass production. Consequently, there is a significant demand for a wideband high-performance BiCMOS PA tailored for advanced mm-wave front ends.

Considerable work has been focused on increasing the operational bandwidth of mm-wave PAs [8,9,10,11,12,13,14,15,16]. Several mm-wave wideband PAs developed in SiGe BiCMOS technologies were reported in [10,11,12,13]. A 60 GHz single-stage, single-ended cascode PA has been designed and fabricated using a 0.13 µm SiGe BiCMOS process, with a focus on maximizing output power while minimizing chip area [11]. The PA features a 12 GHz bandwidth spanning from 54 to 66 GHz, a power gain of 18 dB, and a maximum output power of 14.7 dBm, all within a compact chip area of only 0.3 mm^2^. In reference [10], a transformer-based two-way combining common emitter PA employs a capacitor coupling technique to significantly suppress phase imbalance. The prototype SiGe PA operates under 1.5 V low-voltage conditions, delivering a bandwidth of 42–48.5 GHz, a peak gain of 29.5 dB, and a very flat output 1 dB compression point (OP_1dB_) ranging from 14.5 to 14.8 dBm. However, the saturated output power (P_sat_) is only 16.2 dBm, indicating considerable room for improvement. Distributed architectures offer significant advantages in bandwidth expansion. Lee et al. reported an 18–50 GHz SiGe HBT common-base non-uniform distributed PA [12]. By utilizing a multi-section lumped element artificial transmission line, they achieved optimal load conditions for each active stage across the entire bandwidth. The proposed mm-wave PA reaches a peak P_sat_ of 19.0 dBm and a peak PAE of 19.1% at 32.0 GHz. Nevertheless, this PA occupies a large silicon area of 1.83 mm^2^. To enhance efficiency and output power over a wide frequency range, a traditional transmission line-based impedance inversion technique was applied to a 79 GHz differential Doherty PA in a 130 nm SiGe BiCMOS technology [13]. The proposed amplifier achieves a 1 dB flat bandwidth of 12 GHz, with a P_sat_ of 17.0 dBm and a peak power-PAE of 11.6%. However, achieving this excellent wideband response resulted in a noticeable compromise in the output return loss. Zhao et al. introduced a design approach for interstage and output matching networks in wideband power amplifiers, utilizing wideband inductively coupled resonators and Norton transformations [8]. The prototype PA was fabricated using a 28 nm CMOS process, delivering a P_sat_ of 13 dBm over a 40–67 GHz bandwidth and an OP_1dB_ of 12 dBm without the need for power combining. However, the peak gain is constrained to 13 dB. Additionally, several other wideband PAs based on CMOS technology have also been reported [14,15,16,17,18]. The primary challenge in broadband PA design is achieving a wideband response while balancing key parameters like gain, P_out_, OP_1dB_, and physical size. Although the aforementioned techniques can somewhat broaden the bandwidth, there is still an urgent need for solutions that can significantly extend the bandwidth while maintaining high gain, good gain flatness, high P_out_ and OP_1dB_, as well as a compact footprint.

In this paper, we propose an alternative V-band wideband PA with high gain and high P_out_. The design strategy utilizes a pole-gain tuning approach with dual transmission lines and a common-base (CB) capacitive peaking technique to achieve a broadband gain frequency response. Additionally, it employs a parallel configuration of multiple small-sized transistors to enhance P_out_. The proposed techniques are demonstrated in a two-stage cascode PA using 130 nm SiGe BiCMOS technology. This article is organized as follows. Section 2 discusses the design of the proposed wideband PA, including the overall scheme selection and circuit schematic design. The post-simulation and measurement results that validate the design technique are presented in Section 3. Finally, the paper concludes in Section 4.

## 2. Circuit Analysis and Design

**Figure 1:**
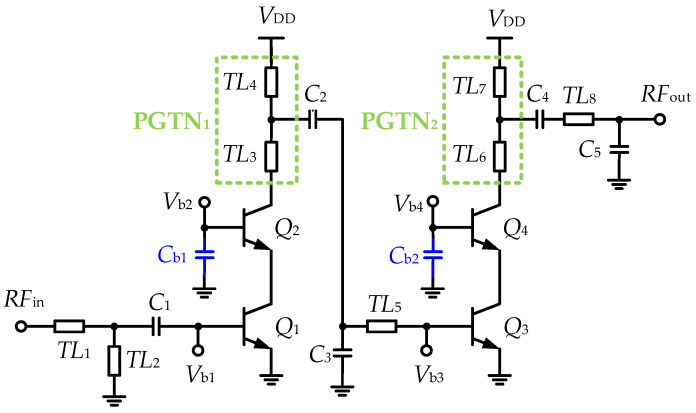
Simplified schematic of the proposed V-band wideband PA.

Figure 1 illustrates the simplified schematic of the proposed two-stage common-emitter-common-base (CE-CB) V-band PA. The CE-CB configuration is chosen for its superior voltage gain, higher breakdown voltage, and improved isolation compared to standalone common-emitter (CE) or CB topologies. Two pole-gain tuning networks (PGTN) with dual transmission lines are applied to both the first and second stages to adjust the dominant poles at different frequencies, thereby achieving a wideband frequency response. The base capacitors *C_b_*_1_ and *C_b_*_2_ are connected to the base terminals of transistors *Q*_2_ and *Q*_4_, respectively, to reduce input parasitic capacitance and enhance power gain, particularly the maximum power gain (MPG) at high operating frequencies. Transmission lines are utilized as inductive passive components due to their smaller dimensions and higher quality factor compared to traditional bulky spiral inductors.

### 2.1. Bandwidth Extension with Pole-Gain Tuning

Given the significant parasitic parameters at mm-wave frequencies, device models have become exceedingly complex, making the optimization of mm-wave amplifiers a considerably challenging task. However, the pole-gain tuning technique has been demonstrated to be an effective approach for enhancing amplifier bandwidth, as it provides great advantages in graphical analysis for bandwidth expansion [19,20]. Generally, the gain-frequency response of an amplifier can be expressed as a function of a complex variable.

This function includes the coefficient *K*, zeros *z_i_*, and poles *p_j_*. Equations (1) and (2), respectively, provide the magnitude and phase responses of the function. As the value of *s* approaches zero, the function value gradually decreases to 0. Conversely, as *s* nears a pole, the function value experiences a substantial increase. The behavior of the gain-frequency function is governed by its zeros and poles. Therefore, the wideband characteristics of the amplifier can be obtained by tuning different dominant poles.

(1) $$\left|H\left(s\right)\right|=K\frac{\prod_{i=1}^{m}\left|s-z_{i}\right|}{\prod_{j=1}^{n}\left|s-p_{j}\right|}$$

(2) $$∠H\left(s\right)=\sum_{i=1}^{m}∠\left(s-z_{i}\right)-\sum_{j=1}^{n}∠\left(s-p_{j}\right)$$

**Figure 2:**
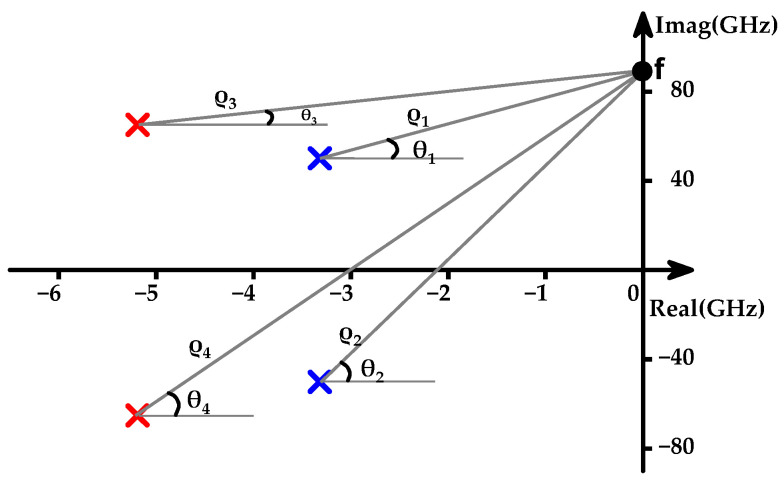
Dominant poles plot for the proposed V-band amplifier.

The dominant poles of the proposed V-band PA with PGTN are plotted in Figure 2, where $\rho_{j}=\left|s-p_{j}\right|$ means the distance from s to pole *j* in the coordinate chart, and $\theta_{j}=∠\left(s-p_{j}\right)$ means the angle from the horizontal. Note that the gain-frequency response function is mainly controlled by poles close to the imaginary axis; poles far away from the imaginary are ignored. Two pairs of complex conjugate poles, *p*_1_ and *p*_2_, and *p*_3_ and *p*_4_, are introduced through PGTNs (PGTN_1_ and PGTN_2_) in the first and second stages, respectively. The *p*_1_ (*p*_2_) govern the low-frequency gain characteristics and are referred to as the low-frequency dominant poles *P*_L_. The pole *p*_3_ (*p*_4_) dominates the high-frequency gain behavior and is designated as the high-frequency dominant pole *P*_H_.

**Figure 3:**
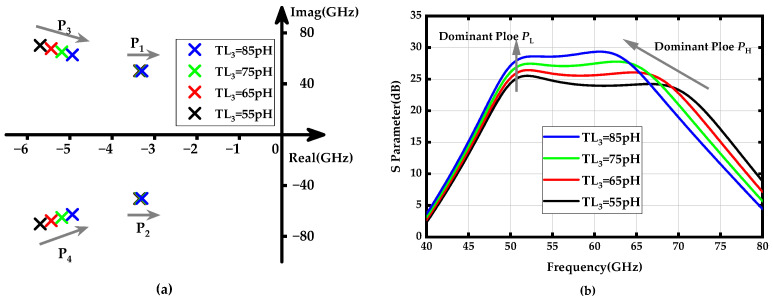
(**a**) Locus of poles *p*_1_ (*p*_2_) and *p*_3_ (*p*_4_) when the *TL*_3_ increases from 55 to 85 pH; (**b**) Gain-frequency response of the proposed amplifier when the *TL*_3_ values vary from 55 to 85 pH.

The PGTN_1_ in the first stage is primarily composed of transmission line inductors *TL*_3_ and *TL*_4_, which are used to generate the high-frequency dominant poles *p*_3_ and *p*_4_. *TL*_3_ is directly connected to the collector of transistor *Q*_2_, isolating the load of the amplifier from the output capacitor. This configuration introduces an initial delay in current reaching other parts of the network, thereby delaying the rise time of the collector current in exchange for improved bandwidth. Figure 3a illustrates the locus of the dominant poles *p*_1_ and *p*_2,_ and *p*_3_ and *p*_4_ as *TL*_3_ increases from 55 pH to 85 pH, while Figure 3b shows the gain-frequency response of the proposed V-band PA under different conditions of *TL*_3_. It can be observed that the high-frequency dominant poles *p*_3_ and *p*_4_ are more sensitive to variations in *TL*_3_ than the low-frequency dominant poles *p*_1_ and *p*_2_. The dominant poles, *p*_3_ and *p*_4_, quickly move toward the imaginary axis and gradually shift to lower frequencies, while *p*_1_ and *p*_2_ approach the imaginary axis much more slowly. This results in a significant reduction in the minimum vector of poles *p*_3_ and *p*_4_, thereby enhancing the amplitude of the high-frequency dominant poles *p*_3_ and *p*_4_. Figure 3b provides a more intuitive physical insight into how the gain-frequency response of the amplifier is influenced by the high-frequency dominant poles.

**Figure 4:**
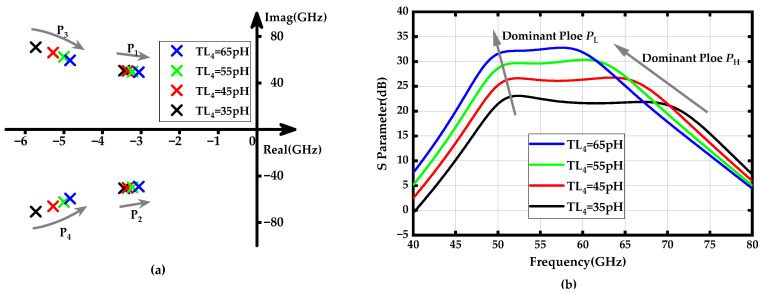
(**a**) Locus of poles *p*_1_ (*p*_2_) and *p*_3_ (*p*_4_) when the *TL*_4_ increases from 35 to 65 pH; (**b**) Gain-frequency response of the proposed amplifier when the *TL*_4_ values vary from 35 to 65 pH.

Similarly, the parallel peaking transmission line inductor *TL*_4_ delays the current flow into its own branch, allowing more of the initial charging current to flow toward the capacitive load. This reduces the rise time and can also extend the bandwidth. Figure 4a depicts the locus of the dominant poles *p*_1_ and *p*_2_, and *p*_3_ and *p*_4_ as *TL*_4_ increases from 35 pH to 65 pH and illustrates the locus of the dominant poles *p*_1_ and *p*_2_, and *p*_3_ and *p*_4_ as *TL*_4_ increases from 55 pH to 85 pH, while Figure 4b displays the PA’s gain-frequency response for different *TL*_4_ values. It can be seen that *TL*_4_ not only adjusts the gain-frequency response of the high-frequency dominant pole but also significantly impacts the amplitude of the low-frequency dominant pole, offering greater flexibility in tuning the amplifier’s frequency response.

**Figure 5:**
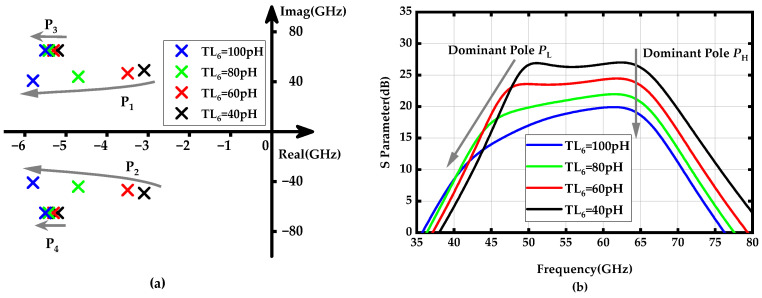
(**a**) Locus of poles *p*_1_ (*p*_2_) and *p*_3_ (*p*_4_) when the *TL*_6_ increases from 40 to 100 pH; (**b**) Gain-frequency response of the proposed amplifier when the *TL*_6_ values vary from 40 to 100 pH.

**Figure 6:**
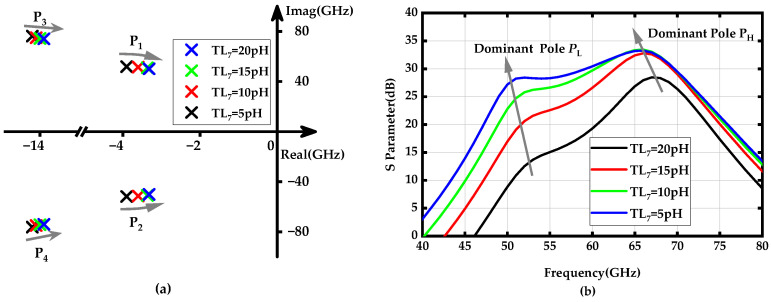
(**a**) Locus of poles *p*_1_ (*p*_2_) and *p*_3_ (*p*_4_) when the *TL*_7_ increases from 40 to 100 pH; (**b**) Gain-frequency response of the proposed amplifier when the *TL*_7_ values vary from 40 to 100 pH.

To achieve further bandwidth expansion, the low-frequency dominant poles *p*_1_ and *p*_2_ should be more flexibly tunable. These poles are established by the PGTN_2_, which consists of the transmission line inductors *TL*_6_ and *TL*_7_, located in the second stage of the amplifier. Figure 5a shows the locus of *p*_1_ and *p*_2_, as well as *p*_3_ and *p*_4_, as *TL*_6_ increases from 40 to 100 pH. It can be observed that the low-frequency dominant poles *p*_1_ and *p*_2_ are significantly affected by changes in *TL*_6_; as the value of *TL*_6_ increases, they rapidly shift away from the imaginary axis and towards lower frequencies. In contrast, although the high-frequency dominant poles *p*_3_ and *p*_4_ also move away from the imaginary axis, they do so at a much slower and more parallel rate, resulting in relatively minor degradation of their amplitude. Figure 5b demonstrates the gain-frequency response verification results for *TL*_6_ variations affecting the low and high-frequency dominant poles. Furthermore, the impact of the transmission line inductor *TL*_7_ on the amplifier’s dominant poles was analyzed, as depicted in Figure 6. These results indicate that both dominant poles shift closer to the imaginary axis, with the lower-frequency poles *p*_1_ and *p*_2_ moving more rapidly. This shift helps mitigate the drawback of enhanced bandwidth at the expense of gain degradation caused by the increase in *TL*_6_.

In summary, the proposed PGTN technique provides an effective approach to extending amplifier bandwidth. Firstly, the generated dominant pole pairs, *p*_1_ (*p*_2_) and *p*_3_ (*p*_4_), remain in the left-half plane, thus ensuring amplifier stability. Secondly, the high-frequency dominant poles *p*_3_ and *p*_4_ generated by PGTN_1_ always move closer to the imaginary axis, significantly enhancing the high-frequency response. Meanwhile, the movement direction and magnitude of the low-frequency dominant poles, *p*_1_ (*p*_2_), can be precisely controlled, providing considerable flexibility in adjusting the amplifier’s overall gain-frequency response.

### 2.2. Common-Base Capacitive Peaking Technique

**Figure 7:**
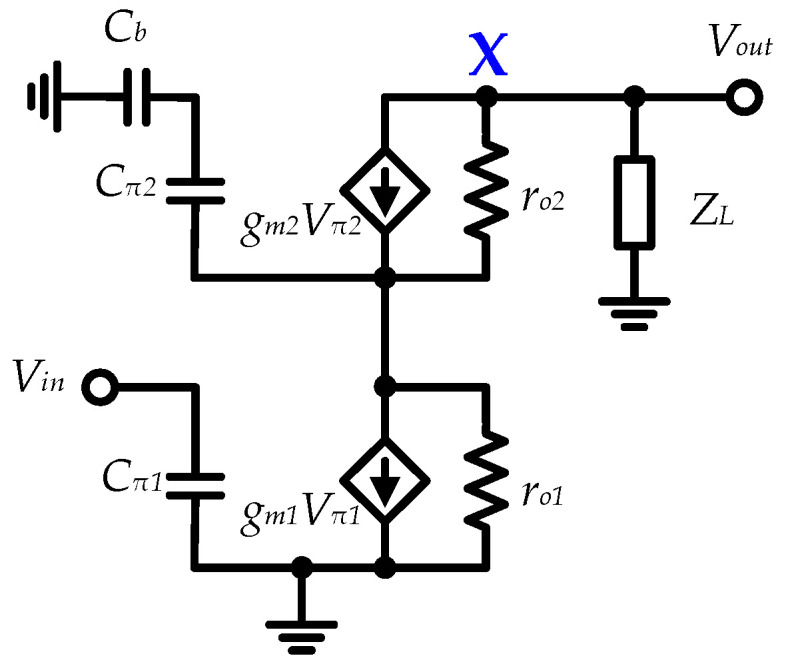
Simplified small-signal equivalent circuit for one-stage CE-CB amplifier.

At millimeter-wave frequencies, significant parasitic effects can degrade the amplifying capability of active transistors. In this work, the capacitive peaking technique is applied to the bases of the CB transistors *Q*_2_ and *Q*_4_ to reduce input parasitic capacitance, thereby enhancing the amplifier’s gain, particularly in the high-frequency band. The simplified small signal equivalent circuit of the one-stage CE-CB amplifier is plotted in Figure 7. Disregarding the emitter junction resistance of the transistor, the gain-frequency response can be approximately calculated as follows:

(3) $$A_{v}=G_{m}\cdot Z_{out}\approx g_{m1}\cdot \left(Z_{x}||Z_{L}\right)$$

(4) $$Z_{out}=Z_{L}||\left[r_{o2}+\left(r_{o2}\cdot g_{m2}+1\right)\cdot \left(\frac{1}{s\cdot C_{s}}||r_{o1}\right)\right]$$

(5) $$C_{s}=\frac{C_{b}\cdot C_{\pi 2}}{C_{b}+C_{\pi 2}}$$

where *G_m_* and *Z_out_* are the total equivalent transconductance and output impedance of the CE-CB amplifier, respectively, *Z_X_* denotes the impedance observed at node *X*, *g_m_*_1_ and *g_m_*_2_ are the transconductances of transistors Q_1_ and Q_2_, while *C_π_*_1_, *C_π_*_2_, *r_o_*_1_ and *r_o_*_2_ are the equivalent input parasitic capacitors and the equivalent output resistors of the transistors Q_1_ and Q_2_, respectively. As inferred from Equations (4) and (5), an increase in *C_b_* will result in a rise in the equivalent output impedance *Z_out_*, thereby enhancing the gain Av.

**Figure 8:**
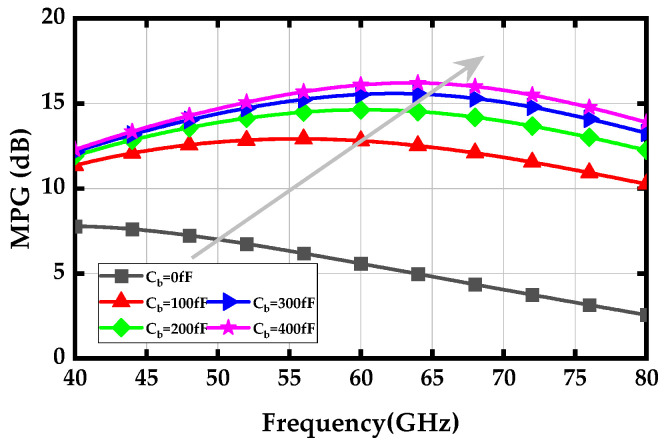
Simulated MPG with variations of base capacitor *C_b_*.

Figure 8 demonstrates the simulated MPG versus variations of the *C_b_*. As the values of *C_b_* increase from 0 to 400 fF, the simulated MPG shows a significant improvement at high frequencies. This enhancement contributes to achieving a flat wideband gain-frequency response, as the transistor’s MPG naturally deteriorates with increasing frequency.

### 2.3. Layout Configuration of Power Stage Transistors

**Figure 9:**
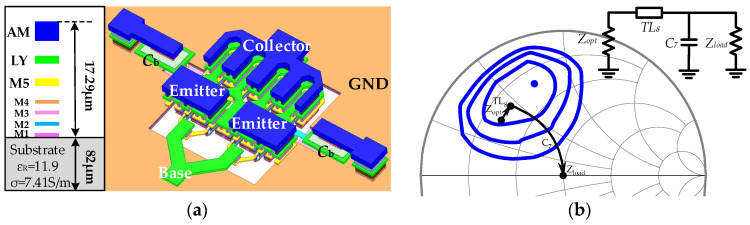
(**a**) The 3D model of power stage transistors with peaking capacitor *C_b_*; (**b**) Simulated power contours of the power stage.

To achieve high P_out_ within the target mm-wave frequencies, large-size transistors are typically used in the power stage. However, this approach can introduce significant parasitic effects, potentially diminishing the output power. These parasitic effects arise not only from the transistors themselves but also from the metal interconnects and vias that connect them. Therefore, optimizing the size and layout of the transistors in the power stage is crucial to minimizing parasitic effects and improving the overall performance of the PA. To construct large-sized power transistor cells with minimal parasitic parameters, a configuration with multiple smaller transistors in parallel is commonly used. As shown in Figure 9a, the power stage employs a parallel and staggered layout of four transistor cells, each with an emitter length of 14 μm. To minimize parasitic capacitance and losses, the interconnects for the emitters and collectors are designed using top-layer metal in an interleaved pattern. Additionally, since the peaking capacitor *C_b_* is directly connected to the CB transistor, it has a significant impact on the overall output impedance of the power stage at mm-wave frequencies. Therefore, *C_b_* is also modeled together with the transistors as an integrated whole.

On the other hand, load-pull simulations were conducted on the output stage at various frequencies to determine the optimal power load impedance. As illustrated in Figure 9b, impedance contours for P_out_ were plotted. Note that the optimal power load impedance often differs significantly from the output matching point. In this design, we selected an impedance of *Z_opt_* = 15.1 + 17.3*j*, which is close to the optimal power load impedance, as the initial value for matching. This choice aimed to strike a balance between P_out_, bandwidth, and output matching. Figure 9b also depicts the impedance transformation path, where the *L*-type network formed by *TL*_8_ and *C*_7_ transforms the 50 Ω load impedance to *Z_opt_*. Furthermore, the source-pull simulation was applied to optimize the output impedance of the first stage, ensuring it delivers sufficient power to drive the power stage.

## 3. Experimental Results

**Figure 10:**
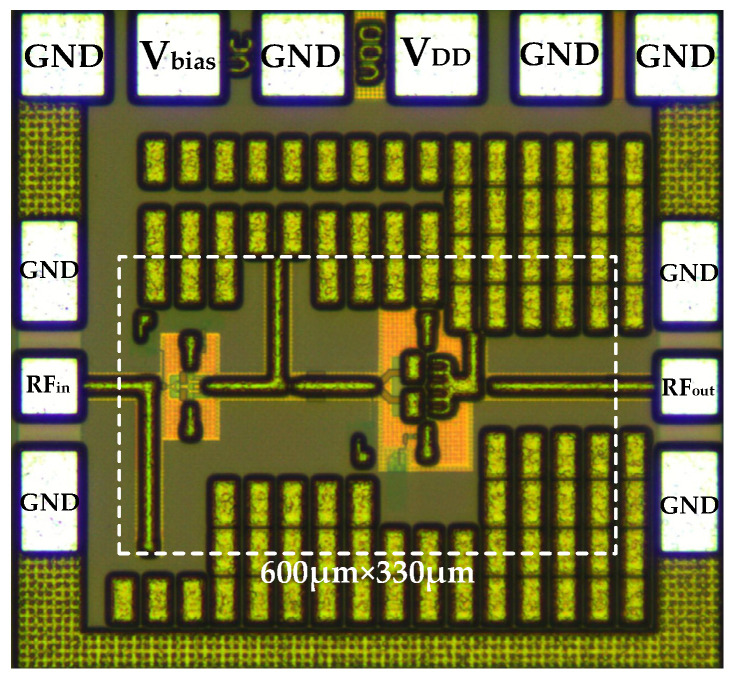
Microphotograph of the proposed wideband PA.

The proposed wideband BiCMOS PA has been designed and fabricated using a 0.13-μm SiGe BiCMOS process, which is a high-performance technology with the compatible 0.13 μm CMOS process. The process provided five thin metal layers and two thick top metal layers labeled AM and LY, respectively. To enhance the quality factor (*Q*), all transmission lines used for impedance matching in the circuit were designed using the thick top metal layer AM. The bottom metal layer M1 served as a ground shield to reduce the parasitic capacitance generated by the transmission lines. Additionally, a series of MIM capacitors were employed in the bypass network to ensure effective power decoupling. Figure 10 shows the die micrograph of the proposed PA. To enhance the amplifier’s performance accuracy, passive components containing transmission lines, capacitors, testing pads, interconnections, vias, and more were meticulously simulated and optimized as a whole using a full-wave 3D electromagnetic field simulator. The whole chip occupied a silicon area of 0.75 mm × 0.76 mm, including all testing pads. The amplifier drew a DC current of 35 mA from a 3.3 V supply voltage, resulting in a power consumption of 115.5 mW.

On-wafer *S*-parameter measurements were carried out over the 45 to 65 GHz frequency range using an MPI TS200-SE probe station with ground-signal-ground (G-S-G) probes having a 150 µm pitch. The measurement setup featured a Keysight PNA-X-N5247B network analyzer with a frequency range of 10 MHz to 67 GHz and a Keysight N6705C DC power analyzer, recognized for its high-resolution capabilities. Calibration was conducted using the standard short-open-load-through (SOLT) method.

**Figure 11:**
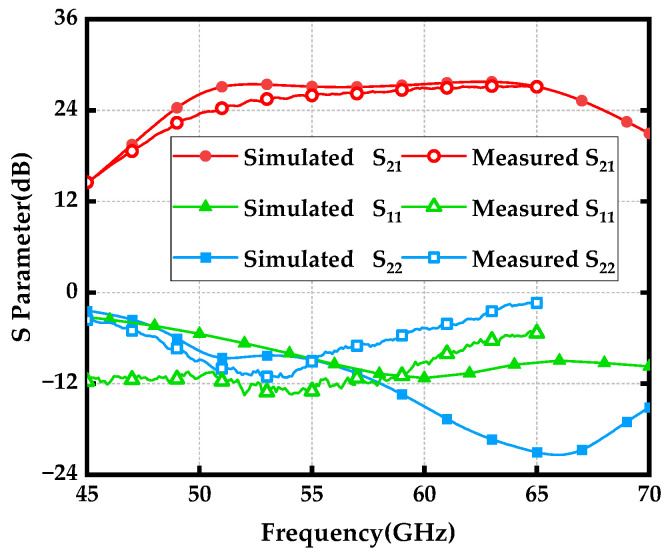
Simulated (solid symbol line) and Measured (hollow symbol line) S-parameters for the wideband PA.

Figure 11 shows the simulated and measured *S*-parameters of the proposed wideband PA, which exhibit a consistent trend. The PA demonstrated an excellent wideband gain response (|S_21_|) with a peak value of 27.3 dB at 64 GHz. The measured −3 dB bandwidth spanned from 52 GHz to 65 GHz. It is worth noting that the high corner frequency of the −3 dB gain bandwidth exceeded 65 GHz, but this could not be captured due to limitations in our testing equipment. Therefore, the PA achieved a −3 dB gain bandwidth of over 13 GHz, with a fractional bandwidth (FBW) exceeding 22.2%. The input return loss (|S_11_|) and output return loss (|S_22_|) are also illustrated in Figure 9, and they were compromised to some extent due to the desired wideband gain response and high output power. Please note that the discrepancies between the simulation and measurement results were primarily due to the foundry adding numerous randomized dummy metal layers during the manufacturing process to pass the Design Rule Check (DRC). These dummy metal layers introduced additional parasitic elements, which degraded the quality factor (*Q*) and effective inductance of the T-matching network.

**Figure 12:**
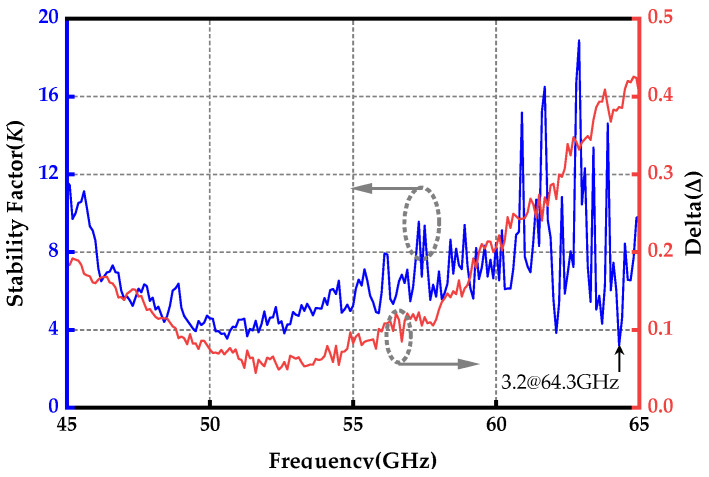
Measured stability factor *K* and delta △.

Figure 12 presents the calculated stability factor *K* and delta Δ derived from the measured *S*-parameters. Across the measured frequency range of 45 to 65 GHz, the stability factor *K* consistently exceeded 3.2, while the delta Δ remained below 1. These results confirm that the proposed wideband amplifier maintained stability under all operating conditions.

**Figure 13:**
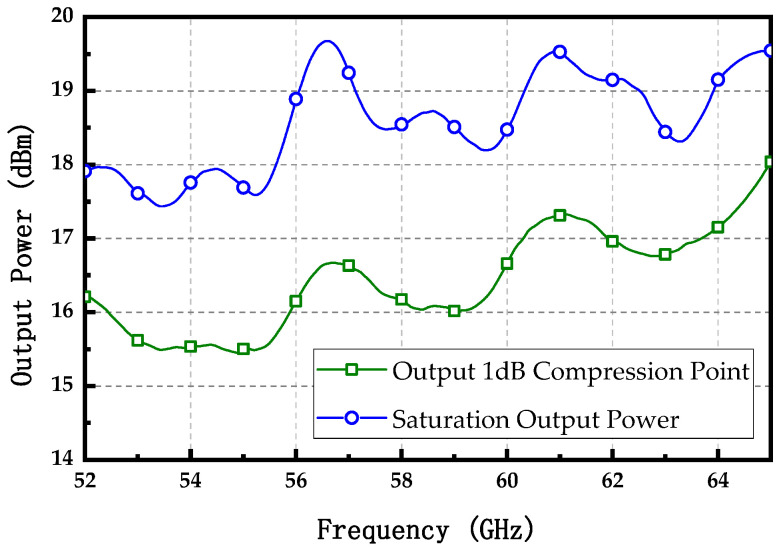
Measured output 1 dB compression point and saturation output power.

The power-handling capability of the wideband amplifier was determined by evaluating the output 1 dB compression point (OP_1dB_) and the saturated output power within the target −3 dB bandwidth. As illustrated in Figure 13, the measured OP_1dB_ and saturated output power varied with operating frequency. Within the specified −3 dB bandwidth frequency range, the OP_1dB_ was observed to range from 15.4 to 18 dBm. In the frequency range of 52 to 65 GHz, the saturated output power varied from 17.4 to 19.7 dBm, demonstrating excellent power flatness with a deviation of less than ±1.15 dBm.

In Table 1, the measured performance of the proposed BiCMOS PA is summarized and compared with the recently demonstrated silicon-based PAs. The proposed PA demonstrated a competitive overall performance, offering a significant bandwidth in extension while maintaining good results in gain, OP_1dB_, and saturated output power.

## 4. Conclusions

A combination of techniques, including pole-gain distribution with PGTN, CB capacitive peaking, and parallel configuration of multiple small-sized transistors, was developed and successfully implemented in a *V*-band PA fabricated in a 130 nm SiGe BiCMOS process. These techniques enable a wideband flat gain-frequency response without compromising other key performance metrics. The PA achieved a peak gain of 27.3 dB, a −3 dB bandwidth exceeding 13 GHz, a maximum P_sat_ of 19.7 dBm, and a maximum OP_1dB_ of 18 dBm while occupying a small footprint of 0.57 mm^2^. This design approach shows significant potential as an effective method for designing wideband amplifiers.

## Figures and Tables

**Table 1:** Performance summary and comparison with recently reported PAs.

Ref.	Technology	Bandwidth (GHz)	FBW (%)	Gain (dB)	P_1dB_ (dBm)	P_sat_ (dBm)	P_dc_ (mw)	Size (mm^2^)
[17]	22 nm SOI CMOS	56.5–63.5 (7)	11.7	22@60 GHz	15.7	18.6	351	0.43
[18]	65 nm CMOS	50.2–59.4 (9.2)	16.78	20.8@54.4 GHz	5.9	13.0	-	0.3
[8]	28 nm CMOS	40–67 (27)	51	13@50 GHz	12	13	-	0.33
[11]	130 nm SiGe BiCMOS	54–66 (12)	20.0	18@61.5 GHz	12	14.7	-	0.30
[10]	130 nm SiGe BiCMOS	42–48.5 (6.5)	14.36	29.5@44 GHz *	14.8	16.2 *	-	1.00
[9]	130 nm SiGe BiCMOS	52–83.4 * (31.4)	46.38 *	17.5@77 GHz	>16	19.1	-	0.68
This work	130 nm SiGe BiCMOS	52–65 (>13)	>22.2	27.3@64 GHz	18	19.7	115.5	0.57

FBW = bandwidth/*f*_c_, where *f*_c_ is the center frequency of −3 dB bandwidth. * estimated from the figure.

## Data Availability

The data presented in this work are available within the article.
